# Toxicity to, oviposition and population growth impairments of *Callosobruchus maculatus* exposed to clove and cinnamon essential oils

**DOI:** 10.1371/journal.pone.0207618

**Published:** 2018-11-16

**Authors:** Luis Oswaldo Viteri Jumbo, Khalid Haddi, Lêda Rita D. Faroni, Fernanda F. Heleno, Frederico G. Pinto, Eugênio E. Oliveira

**Affiliations:** 1 Departamento de Entomologia, Universidade Federal de Viçosa, Viçosa, MG, Brazil; 2 Embrapa Tabuleiros Costeiros, Aracaju, SE, Brazil; 3 Departamento de Engenharia Agrícola, Universidade Federal de Viçosa, Viçosa, MG, Brazil; 4 Departamento de Química, Universidade Federal de Viçosa, Campus Rio Paranaíba, MG, Brazil; Institut Sophia Agrobiotech, FRANCE

## Abstract

The use of plant essential oils has been shown to efficiently control insect pests of stored beans, significantly reducing the threats associated with synthetic insecticides. Here, we evaluated the potential of applications of essential oils of clove, *Syzygium aromaticum* L., and cinnamon, *Cinnamomum zeylanicum* L., to control *Callosobruchus maculatus*, considered as one of the most cosmopolitan pests of stored beans. Using four combinations of couples (i.e., unexposed couples, exposed females, exposed males, and exposed couples), we also evaluated how sublethal exposure to these essential oils impacted *C*. *maculatus* oviposition. Bioassays results revealed that both essential oils exhibited insecticidal activities similar to the synthetic pyrethroid insecticide deltamethrin. Furthermore, oil dosage increments proportionately decreased the growth rate and reduced the losses in bean weight caused by cowpea weevils, and offspring emergence was almost abolished when parents were exposed to the LD_20_ of each essential oil. Finally, significant oviposition impairments were perceived only in couples where females were exposed (i.e., females exposed and exposed couples) to the LD_20_ of cinnamon and clove essential oils. Thus, by exhibiting similar insecticidal activities as synthetic insecticides and by significantly affecting the oviposition of sublethally exposed *C*. *maculatus* females, the cinnamon and clove essential oils represent valuable tools with potential of integration into the management of *C*. *maculatus* infestations.

## Introduction

Plant essential oils have gained a reputation as being potentially bioactive compounds against many insect species, which has portrayed them as safer tools in terms of the environment and human health [1–6]. Despite the potential of essential oils to control pests of stored products, few studies have addressed the physiological and biological responses of stored product pests when the exposure occurred at sublethal levels.

It has been well recognized that stored product pests when sublethally exposed to synthetic insecticides can exhibit not only detrimental (what is somehow expected), but under certain circumstances also positive responses on their physiology and behavior [7–11]. Although the mechanisms explaining such sublethal responses (i.e., positive or detrimental) are still not well understood, it has been described that individuals sublethally exposed to synthetic insecticides show alterations in relevant life traits (e.g., the development time, longevity, fertility, fecundity, immune capacities, locomotion, navigation, sexual communication, oviposition and feeding) [7]. It is worthy to note, however, that similar life trait alterations seem to be elicited by sublethal exposure to botanical insecticides, which in their turn can trigger insect responses that either increase [12–16] or compromise the efficacy of these alternative control tools [9, 17, 18].

Among the essential oils that have been shown to adequately control insect pests, the oils extracted from clove, *Syzygium aromaticum* (L.), and cinnamon, *Cinnamomum zeylanicu*m (L.), plants have drawn particular interest because of their promising insecticidal activities against various pests of stored products such as the maize weevil *Sitophilus zeamais* and the red flour beetle *Tribolium castaneum* [2, 12–16]. In insects, these essential oils have neurotoxic action both as fumigants and or contact insecticides and there metabolites can act upon variety of molecular targets including inhibition of acetylcholinesterase or disturbing the functions of GABAergic and aminergic systems [19].

The cowpea weevil, *Callosobruchus maculatus* Fabricius (Coleoptera: Chrysomelidae: Bruchinae), is a cosmopolitan pest of legume seeds and is among the most serious pests of stored products in tropical countries [20–22]. The insect larvae represent the most destructive stage, as adult cowpea bruchid do not feed [23, 24]. However, as the availability of a specific host is highly discontinuous and because these adult insects have to live in hosts that are normally treated with insecticides [25–27], these insects might have to face insecticidal sublethal exposures prior to deciding where they are going to lay eggs.

Most of the cowpea, *Vigna unguiculata* (L.) Walp, production occurs in tropical countries with high contribution of small farmers. In this context, the control of *C*. *maculatus*, when accomplished, is mainly achieved by the application of a small group of synthetic molecules (e.g., phosphine and pyrethroid insecticides, such as deltamethrin). Dependence on a small group of synthetic molecules raises the risk of selecting resistant populations as well as increases the hazard risks to human health and to the environment [2, 28].

Thus, it is urgently needed to develop alternatives to the chemical control of *C*. *maculatus* that not only can reduce the concerns outlined above but also can be prone of embracing the actual trend in developing new botanical-derived insecticides based on the inclusion of the active ingredient (i.e. EOs) in stable delivery systems (nanoparticles, nanoemulsion, etc) [29, 30]. Therefore, we investigated the chemical composition of clove and cinnamon essential oils and evaluated whether these oils would adequately control *C*. *maculatus*. We also characterized the biological responses (e.g., oviposition, offspring emergence and population growth) of *C*. *maculatus* exposed to sublethal amounts of each type of essential oil.

## Materials and methods

### Insect rearing

The original population of *C*. *maculatus* was field-collected from small farms in the Viçosa region (Minas Gerais State, Brazil) during the year 2015, and the population was maintained on pest- and insecticide-free cowpea beans under laboratory conditions (27 ± 2°C, 75 ± 5% RH, 12 h scotophase). The bean grains had a water content of 12% and were offered *ad libitum*. The farm-owners gave permission to collect samples of *C*. *maculatus* from their fields and since *C*. *maculatus* is not an endangered or protected species in Brazil, no specific permissions were required for such collection.

### Extraction and chemical characterization of essential oils

Locally purchased cinnamon bark and dried flower buds of clove were used for essential oil extraction, as described by [31]. Briefly, the primary material was ground and sieved to obtain a fine powder (less than 1 mm) that was extracted at room temperature by constant percolation with hexane, followed by hydrodistillation for 6 h. Then, the distillate was extracted twice with dichloromethane and dried over anhydrous sodium sulfate. The distilled oils were stored in airtight screw-capped vials at -10°C until use.

The components of the cinnamon and clove essential oils were determined by gas chromatography-mass spectrometry (GCMS-QP2010, Shimadzu). The separation was done on a capillary column of fused silica (30 m × 0.22 mm) with stationary phase RTX5 (0.25-μm-thick film). The initial column temperature was 60°C for 2 min, followed by increase of 3°C min^-1^ up to 240°C, and this temperature was maintained for 15 min. The temperatures of the injector and detector were maintained at 220°C and 240°C, respectively.

The carrier gas was helium with a flow of 1.8 mL min^-1^. Samples were diluted in dichloromethane and injected in a 1.0 μL split ratio of 1:20. Data acquisition was made in full-scan mode, with a scanning range between 29 and 400 m/z. Experimental mass spectra were compared with known mass spectra (The National Institute of Standards and Technology (NIST 14) Mass Spectra Library, 2017). The arithmetic index (AI) was calculated according to [32], using the retention times of the essential oil compounds and a homologous series of C_8_-C_26_ n-alkane standards following the formula: AI (x) = 100 P_z_ + 100[(t (x) − t (P_z_))/t (P_z+1_) − t (P_z_))]; where x: compound at time t; P_z_: alkane before x; and P_z+1_: alkane after x. The relative percentage of each compound was calculated by the integral ratio of its respective peak area and the total area of all the compounds of the sample. The calculated AI for each compound was compared with values reported in the literature [32].

### Insecticidal activity

We conducted dose-mortality bioassays to determine the lethal doses of the cinnamon and clove essential oils to adult *C*. *maculatus*. These bioassays followed procedures previously described elsewhere [33]. Briefly, each essential oil was pure (i.e., without diluents) and was applied using a 25-μL microsyringe (Hamilton, Reno, NV, USA) to 50 g of beans that were placed in 0.8-L glass jars. After the application, the jars were manually shaken for 60 s to ensure a complete distribution of the essential oils. Twenty unsexed 1-2-day-old *C*. *maculatus* adults were placed in each jar, and the jars were sealed with a fine porous cloth to allow ventilation; jars were kept under controlled conditions (27 ± 2°C, 75 ± 5% relative humidity, 12 h scotophase). The insect mortality was recorded after a 24-h exposure period. Insects were considered dead if they did not respond to fine paintbrush stimuli (i.e, two subsequent touches in 2 min intervals). Five doses of each essential oil were tested in the bioassays (e.g., *Cinnamon*: 20, 60, 120, 160 and 200 μl kg^-1^. *Clove*: 20, 40, 80, 120, 160 μl kg^-1^). Five replications were used per dose, and the control treatment did not receive any oil application. As a positive control, we used the pyrethroid insecticide deltamethrin (25 g L^-1^; EC; Bayer Crop Science, SP, Brazil) diluted in distilled water to obtain the desired doses (e.g., 64, 72, 80, 88, 96, 104 μl a.i kg^-1^). The application and the conditions of bioassays were similar to those described for the essential oils.

### Effects of essential oils on the biological development and bean-mass losses

#### Effects on instantaneous rate of population growth (*r*_*i*_) and bean-mass losses

The instantaneous rate of increase (*r*_*i*_) test was carried out in 0.8-L glass jars, where 20 unsexed 1-2-day-old adults of *C*. *maculatus* were allowed to colonize 50 g of beans treated with an essential oil based on the concentration-mortality results previously obtained (see Results section). We measured the instantaneous rate of increase (*r*_*i*_) of groups of *C*. *maculatus* that were subjected to different sublethal exposures to cinnamon (LD_20_ = 106.2, LD_40_ = 123.0, LD_60_ = 139.4, LD_80_ = 161.4 μL kg^-1^ of bean) and clove (LD_20_ = 48.6, LD_40_ = 67.6, LD_60_ = 90.2, LD_80_ = 125.8 μL kg^-1^ of bean) essential oils. Five replicates were used for each combination of concentration and essential oil. All the glass jars were maintained at 27 ± 2°C, 75 ± 5% relative humidity and 12 h scotophase. The control treatment did not receive any essential oil application. The number of F_1_ insects was counted after 45 days, and the instantaneous rate of increase for each population was calculated as follows: *r*_*i*_ = *ln (N*_f_*/N*_i_*)/*Δ*t*, where *N*_f_ is the final number of observed adults, *N*_i_ is the initial number of *C*. *maculatus*, and Δ*t* is the duration of the experiment [34].

The grain masses provided for insect colonization were weighed at the start (day 0) and at the end (day 45) of the bioassays to calculate the percentage of grain loss.

#### Effects on average and cumulative emergence

The bioassays for the average emergence were conducted using the same experimental procedures described for the instantaneous rate of increase (*r*_*i*_). The progeny formed by the adult *C*. *maculatus* emerging from the beans were assessed in two days intervals starting from the 20^th^ day after treatments began until no adult emergence was observed (i.e., 20 days after emergence of the 1^st^ adults). After each assessment, the emerged adults were removed.

### Effects of sublethal exposure to essential oils on the *C*. *maculatus* oviposition

Newly emerged (< 48 h old) groups of *C*. *maculatus* adult males and females were exposed separately to clove- and cinnamon-essential-oil-treated beans at the LD_20_ values for clove (i.e., 48.6 μL kg^-1^ of beans) and for cinnamon (i.e., 106.2 μL kg^-1^ of beans) essential oils. After a 24-h exposure period, we paired *C*. *maculatus* couples in four combinations (i.e., unexposed couple, exposed female, exposed male, and exposed couple) and allowed each couple to oviposit in 20 g of untreated beans. At 3-day intervals, the couples were moved to new 20-g bean masses, and this process was repeated for a total period of 9 days. The number of oviposited eggs was assessed under microscope after 3, 6 and 9 days. Twenty couples were used for each treatment combination.

### Statistical analyses

Dose-mortality data were subjected to probit analysis [35], and 95% confidence intervals for toxicity ratios were estimated following [36]; the values were considered significant if the range did not include the value 1. Regression analyses were performed to detect trends in cumulative and average emergence that resulted in each treatment over time. Regression analysis was performed using the curve-fitting procedure of Sigma Plot 12.0. The regression model was chosen based on parsimony, lower standard errors, and steep increases in R^2^ with increases in model complexity. The regression models for each treatment were considered different from each other if the confidence limits of their parameters did not overlap. We also conducted linear regression to assess the effects of increasing lethal exposure to essential oils on the *r*_*i*_ and grain-mass losses of *C*. *maculatus*. The data on the number of eggs used in each treatment combination were submitted to repeated measures ANOVA. The assumptions of normality and homogeneity of variance were tested for all parameters, and no data transformations were necessary (PROC UNIVARIATE, SAS Institute Inc., Cary, NC, USA).

## Results

### Chemical composition of the essential oils

The chemical analyses showed that the two main components of cinnamon and clove essential oils were eugenol and β-caryophylene (Table 1). However, the cinnamon essential oil additionally contained a wide range of other compounds in smaller amounts, including acetyleugenol, benzyl benzoate, linalool, cinnamyl acetate and cinnamaldehyde.

**Table 1:** Chemical composition of clove and cinnamon essentials oils.

Constituents	Arithmetic index				Concentration		
	*S*. *aromaticum*		*C*. *zeylanicum*		*S*. *aromaticum*	*C*. *zeylanicum*	
	a	b	a	b	(%)		
eugenol	1363	1356	1364	1356	87.4	73.1	
*β*-caryophylene	1415	1417	1414	1417	11.5	7.7	
*α*-humulene	1447	1452	1447	1452	1.1	0.4	
α-pinene	-	-	931	932	-	0.7	
α-phellandrene	-	-	1004	1002	-	0.3	
p-cymene	-	-	1022	1020	-	1.0	
limonene	-	-	1026	1024	-	0.5	
eucalyptol	-	-	1028	1026	-	0.7	
linalool	-	-	1100	1095	-	2.6	
E-cinnamaldehyde	-	-	1268	1267	-	2.3	
methyleugenol	-	-	1405	1403	-	0.6	
cinnamyl acetate	-	-	1444	1443	-	2.5	
acetyleugenol	-	-	1528	1521	-	3.6	
caryophyllene oxide	-	-	1576	1582	-	0.5	
benzyl benzoate	-	-	1760	1759	-	3.4	

^a^ calculated

^b^ tabulated.

### Insecticidal activity

The mortality levels obtained in the dose-mortality bioassays were satisfactorily described by the probit model [goodness-of-fit tests exhibiting low χ^2^-values (<10) and high *P*-values (>0.05)]. The toxicity ratios (TR) were estimated relative to the LD_50_ for deltamethrin. The toxicities of the clove and cinnamon essential oils were similar to the pyrethroid-based insecticide deltamethrin (Table 2).

**Table 2:** Toxicity of clove and cinnamon essential oils and deltamethrin on adults of *Callosobruchus maculatus*.

Insecticide	Slope ± SD	LD_20_ (95% FL)	LD_40_(95% FL)	LD_50_ (95% FL)	LD_60_ (95% FL)	χ^2^	*P*	TR_50_ (95% CL)
Clove	4.0 ± 0.32	48.6 (42.0–54.0)	68.0 (62.0–74.0)	78.2 (71.6–84.8)	90.0 (84.0–98.0)	5.25	0.15	0.94 (0.9–1.0)
Cinnamon	9.3 ± 1.02	106.4 (96.0–114.0)	124.0 (116.0–130.0)	131.0 (124.0–137.0)	138.0 (132.0–146.0)	3.90	0.14	1.56 (1.5–1.6)
Deltamethrin	13.9 ± 1.25	72.8 (68.8–76.0)	80.0 (76.8–83.2)	83.7 (80.6–86.6)	87.2 (84.0–90.3)	9.91	0.08	1.00 (0.9–1.0)

SD standard deviation; LD: Lethal dose (μL kg^-1^); FL = Fiducial limits; χ^2^ **=** *Chi*-square for lack-of-fit to the probit model, and *P* = Probability associated with the *chi*-square statistic; TR_50_ = Toxicity ratio determined by LD_50_ of each the essential oil /LD_50_ of deltamethrin; CL = Confidence limits of TR_50_.

### Effects of essential oils on the biological development and bean-mass losses

#### Effect on instantaneous rate of population increase (*r*_i_) and bean weight loss

**Fig 1:**
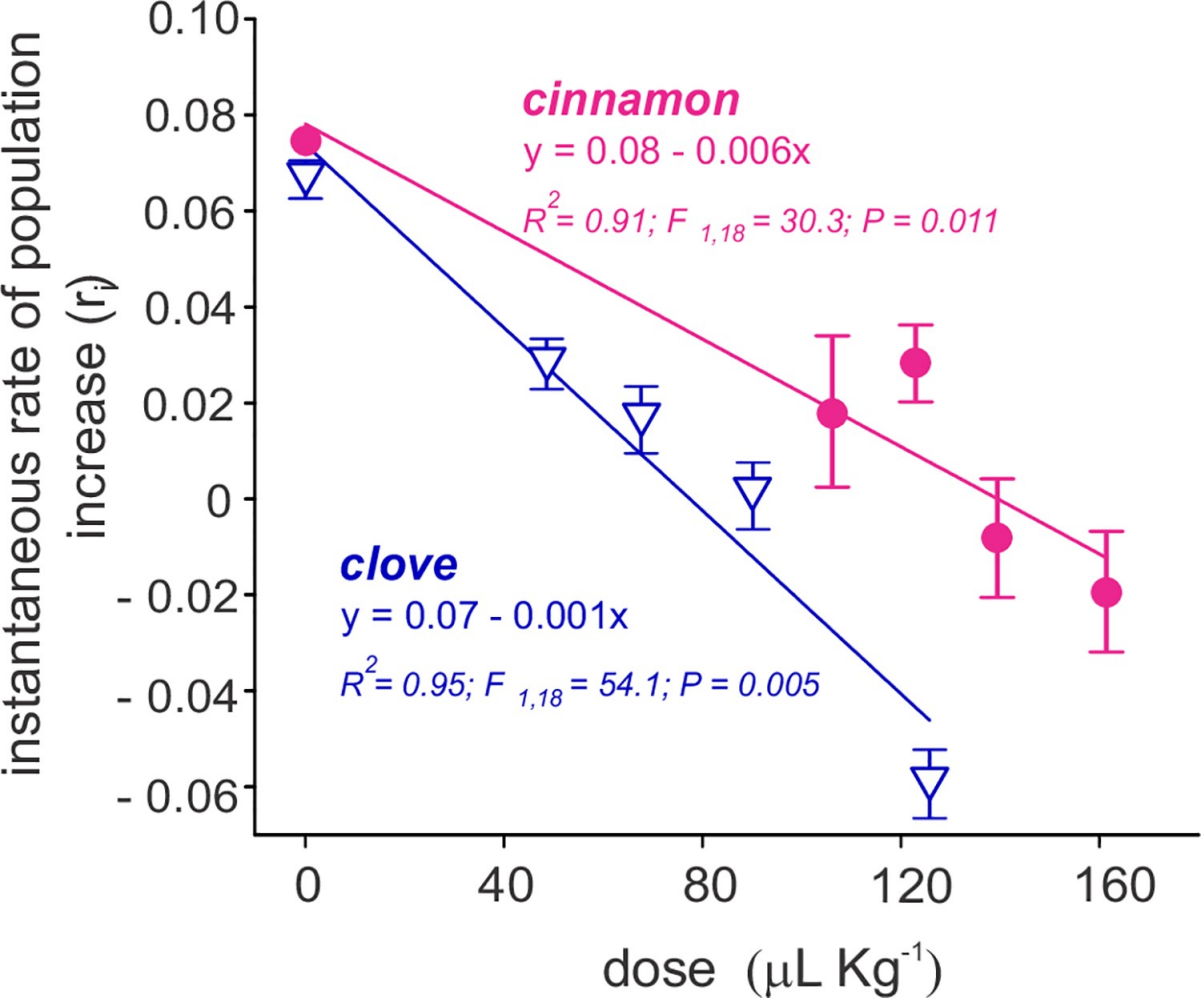
Instantaneous rate of population increase of *C*. *maculatus* exposed to clove and cinnamon essential oils. The symbols represent the means of five replicates of the LD_0_ (control), LD_20_, LD_40_, LD_60_ and LD_80_ for each oil. The doses are expressed in μL of essential oil/kg beans. The vertical bars represent the SD.

**Fig 2:**
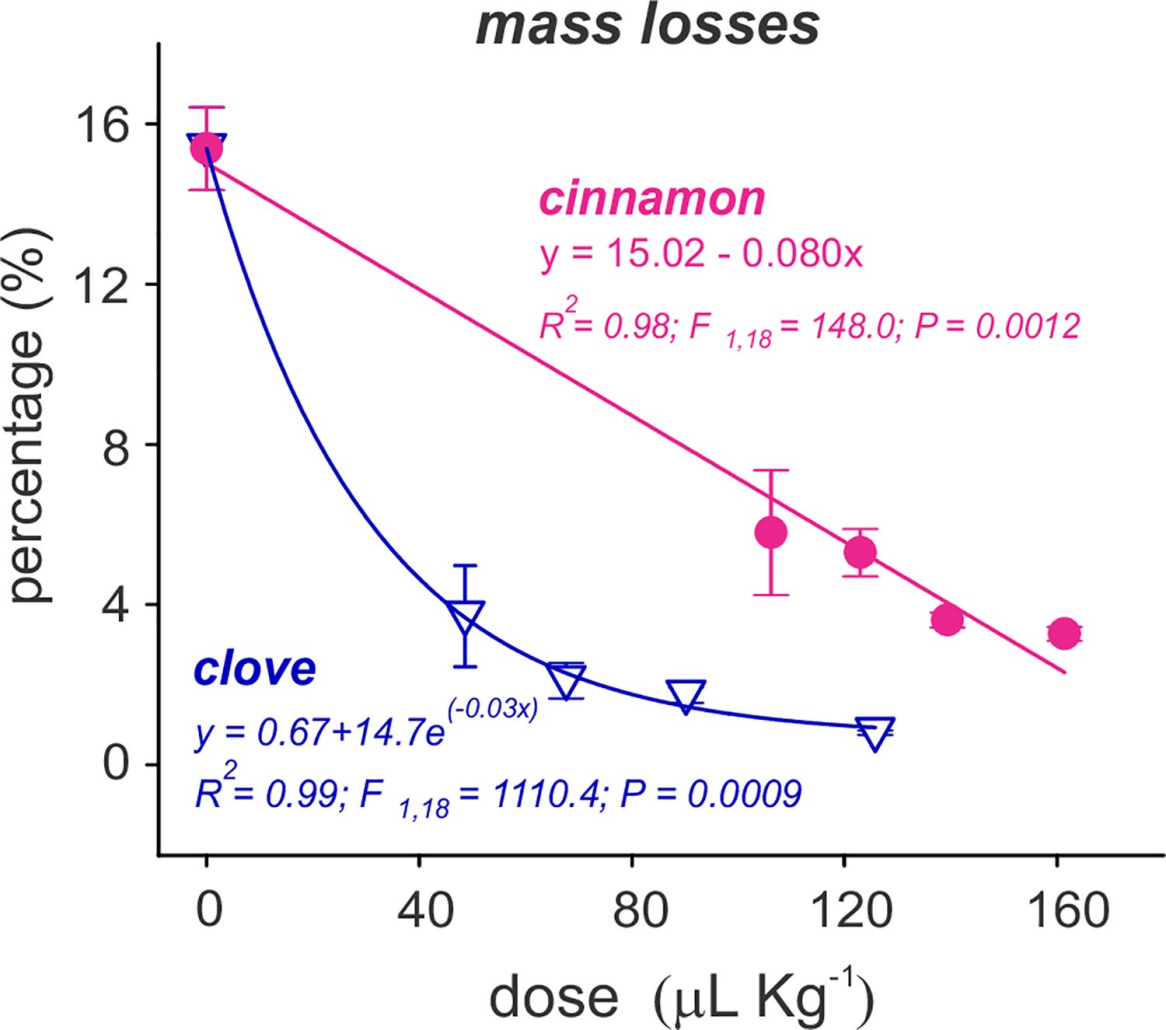
Bean weight losses caused by *C*. *maculatus* exposed to clove and cinnamon essential oils. The symbols represent the means of five replicates of the LD_0_ (control), LD_20_, LD_40_, LD_60_ and LD_80_ for each oil. The doses are expressed in μL of essential oil/kg beans. The vertical bars represent the SD.

The instantaneous rate of population increase (*r*_*i*_) decreased in a dose-dependent manner for the two essential oils used (Fig 1). The extinction stage (negative *r*_*i*_) was reached when the *C*. *maculatus* insects were in contact with concentrations equal to or higher than LD_60_ for clove (i.e., 67.6 μL kg^-1^ of bean) and cinnamon (i.e., 139.4 μL kg^-1^ of bean) essential oils. Similar trends were observed for the bean mass losses, where the LD_20_ concentrations of clove (*F* = 21.3; *P* < 0.001) and cinnamon (*F* = 69.8; *P* < 0.001) essential oils significantly reduced grain loss when applied as a contact treatment (Fig 2). These losses were reduced from approximately 15%, when the beans were incubated with *C*. *maculatus* insects in the absence of essential oils, to less than 6%, when the bean was treated with clove and cinnamon essential oils.

Positive correlations were observed between the instantaneous rate of increase of *C*. *maculatus* and the mass losses in grain masses treated with clove *(R*^*2*^ = 0.72; *P* < 0.001) and cinnamon *(R*^*2*^ = 0.83; *P* < 0.001) essential oils, between the instantaneous rate of increase and the total number of emerged adult of *C*. *maculatus* for clove *(R*^*2*^ = 0.75; *P* < 0.001) and cinnamon *(R*^*2*^ = 0.85; *P* < 0.001) essential oils, and between the total number of emerged adult of *C*. *maculatus* and the grain mass losses for clove *(R*^*2*^ = 0.98; *P* = 0.03) and cinnamon *(R*^*2*^ = 0.99; *P* < 0.001) essential oils. All of the fitting parameters for the curves in Figs 1 and 2 are presented in supplementary S1 Table.

#### Effects on average and cumulative emergence

**Fig 3:**
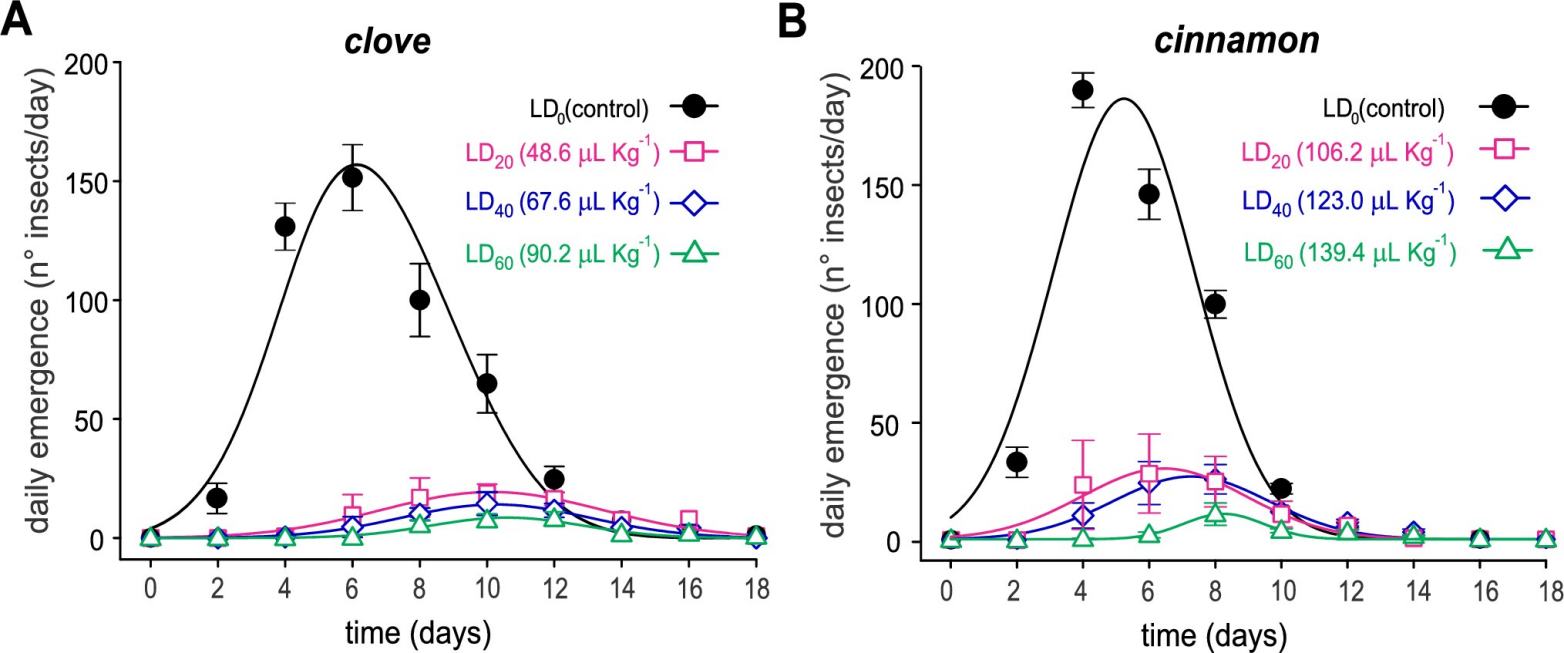
**Average emergence of *C*. *maculatus* exposed to clove (*A*) and cinnamon (*B*) essential oils.** The symbols represent the means of four replicates of the LD_0_ (control), LD_20_, LD_40_, and LD_60_ for each oil. The doses are expressed in μL of essential oil/kg beans. The vertical bars represent the SD.

**Fig 4:**
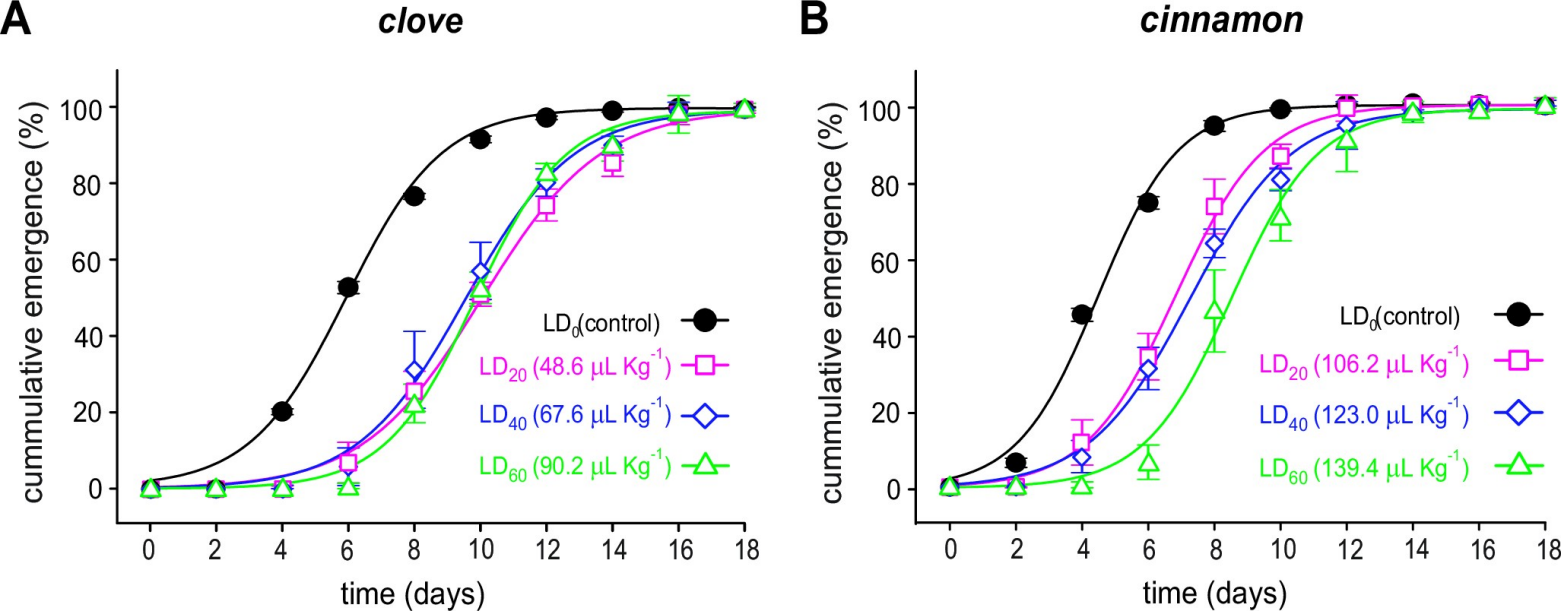
**Normalized cumulative emergence of *C*. *maculatus* exposed to clove (*A*) and cinnamon (*B*) essential oils.** The symbols represent the means of four replicates of the LD_0_ (control), LD_20_, LD_40_ and LD_60_ for each oil. The doses are expressed in μL of essential oil/kg beans. The vertical bars represent the SD.

The average emergence of new *C*. *maculatus* insects was negatively and severely impacted after treatment by either clove or cinnamon essential oil, as observed in the differences between the emergence curves (Fig 3). Treatments with concentrations starting at the LD_20_ for both essential oils resulted in a near abolition of emergence. Moreover, the total emergence of *C*. *maculatus* was significantly delayed by treatments of almost all concentrations of the two essential oils when compared to the control, as shown by the lack of overlap between the cumulative emergence curves (Fig 4). All of the fitting parameters for the curves in Figs 3 and 4 are presented in supplementary S2 and S3 Tables.

### Effects of sublethal concentrations of essential oils on *C*. *maculatus* oviposition

**Fig 5:**
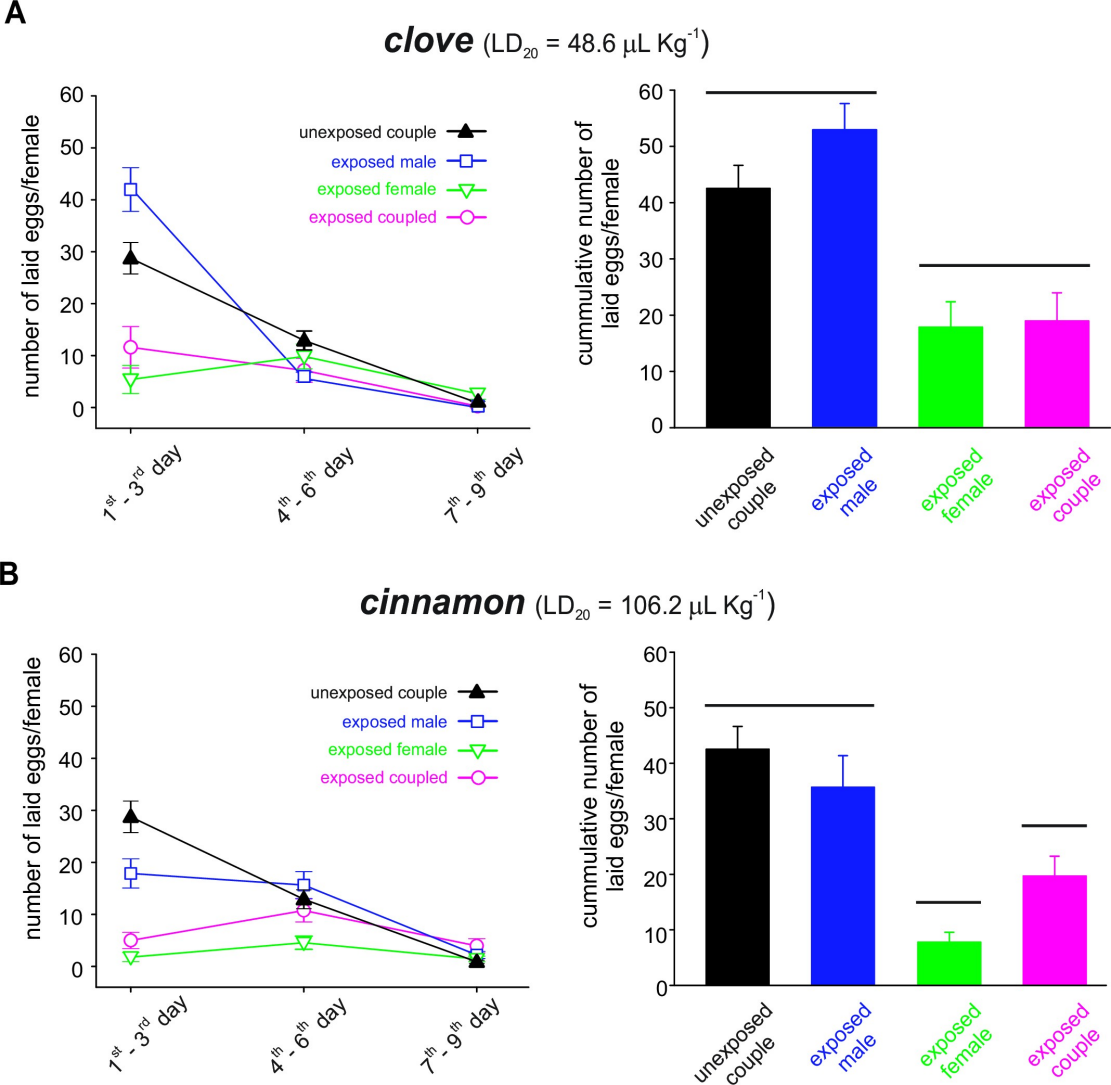
Effects of sublethal exposure to essential oils on *C*. *maculatus* oviposition. Number of eggs of *C*. *maculatus* females that were sublethally exposed to clove (*A*) or cinnamon (*B*) essential oils and coupled with essential oil-treated or essential oil-untreated partners. The symbols represent the means of 20 replicates (± SD) for the number of eggs laid by females of *C*. *maculatus* at three-day intervals (left panels) and the cumulative number of eggs (right panels) laid on cowpea bean masses during the first 9 days of adulthood. On the right panels, the treatments grouped by the same horizontal line did not differ according to a Tukey HSD test (*P* < 0.05).

The results of repeated measures ANOVA showed that there was a significant interaction (Wilks’ lambda = 11.77; *df* = 6; *P* < 0.001) between oil types, couple combinations and evaluation times (Fig 5). The sublethal exposure to clove and cinnamon essential oils mediated the effects on *C*. *maculatus* oviposition, resulting in significant (*F* = 25.1; *df* = 3; *P* < 0.001) differences in the total number of eggs among the four combinations of treatments. When only females were exposed (i.e., exposed females or exposed couples) or when both females and males were exposed (i.e., exposed couples), the total number of laid eggs decreased dramatically in comparison with both the untreated couples and the couples where only males were treated. The two essential oils showed significantly (*F* = 4.19; *df* = 1; *P* = 0.04) different inhibiting effects on oviposition, and this difference was more evident in the oviposition of couples where only females were treated, as the decrease was more important for cinnamon compared to clove and untreated couples. Moreover, the differences between the effects of sublethal exposure to both essential oils were significantly (*F* = 136.2; *df* = 2; *P* < 0.001) higher during the first 3 days of the oviposition period.

## Discussion

Plant essential oils are among the most interesting options for cheaper, safer and eco-friendly replacements (or to be used as adjuvants) for synthetic insecticides [2–4, 6]. Here, we demonstrated that applications of clove and cinnamon essential oils not only adequately controlled *C*. *maculatus* on stored cowpea beans but also were capable of reducing the oviposition and population growth of *C*. *maculatus* even at sublethal dosages.

Essential oils, such as clove and cinnamon oils, are very complex natural mixtures and can contain various compounds at different concentrations with two or three major components that will determine the biological properties of the essential oil [37]. However, synergistic effects between the components of essential oils have been frequently reported in previous studies [38–43]. Our chemical analyses of cinnamon and clove essential oils revealed that their primarily components were eugenol (>70.0%), followed by the sesquiterpene β-caryophyllene (between 7.0 and 12%). These results are in concordance with previous studies that reported similar compositions [33, 37, 44–48]. It is worth noting that cinnamon essential oil, despite its major components, also contained a range of other compounds, including acetyleugenol, benzyl benzoate, linalool, cinnamyl acetate and cinnamaldehyde (between 2 and 4%), lending more evidence to the hypothesis that essential oil biological activities may be shaped by the potential synergistic and antagonistic interactions among all these molecules and not only by major essential oil compounds [37, 49].

Several studies have reported the insecticidal toxicity of clove and cinnamon essential oils and their primary compounds that successfully control stored product pests [9, 18, 26, 33, 50–52] and other insects [48, 53–56]. The vast majority of these investigations have attributed these essential oil insecticidal activities to their major constituents (i.e., eugenol and β-caryophyllene), as these compounds are known to act on the insects’ nervous system by disturbing the functions of GABAergic [57, 58] and aminergic [59–61] systems and by inhibiting the actions of acetylcholinesterase [62–64].

Negative effects on developmental traits, such as rates of growth and progeny emergence of bruchid insects such as *C*. *maculatus*, have been reported for various essential oils and their components [22, 23, 26, 52, 65–69]. The present investigation demonstrated that treating cowpea bean masses with sublethal dosages (i.e., as lower as their LD_20_) of these essential oils leds to significant decreases in the *C*. *maculatus* instantaneous rates of population growth and the bean mass losses, and the application of the essential oils almost abolished *C*. *maculatus* offspring emergence. Such biological impairments caused by clove and cinnamon essential oils on bruchids can be the result of the direct mortality of adults, repellency, oviposition deterrence or progeny and growth inhibition [51]. However, our oviposition results (i.e., females sublethally exposed to these essential oils decreased their ability to lay eggs even when they were offered to mate with untreated sexual partners in untreated bean masses) revealed an even more complex scenario and potential effects on sexual fitness (e.g., locomotory activities, mating behavior) or on the physiological basis of oviposition (e.g., respiratory activities, oogenesis or hormonal disruption) may also contribute to the reduced performance of essential oil sublethally exposed insects. For instance, similar physiological impairments (e.g., repellence, emergence inhibition, altered respiratory activities and transgenerational behavior changes) have been reported in stored product pests (e.g., *S*. *zeamais*) exposed to essential oils of cinnamon and clove [9, 13, 17, 18].

Furthermore, as biosynthesis and release of mating signals as well as the production of eggs may be influenced by atmospheric volatiles and gases [70, 71],. plant extracts, such as terpenes, can influence the site-choice of egg-laying female insects [70]. Although future experiments are required to isolate the effects of the exposure to essential oils on the physiological basis of oviposition and on the mating behavior, a potential energy trade-off between the detoxification process and oogenesis might be an explanation for the inhibited oviposition observed here [72–74].

In our study we have used drops of pure essential oils on bean masses and although such technique showed good biological activities on *C*.*maculatus* under laboratory conditions and may have potential applications at small farmer’s level, this delivery system may suffer from draw backs inherent to the volatile nature of essential oils in larger storage facilities. In fact, rapid biodegradation of these compounds due to their poor physicochemical stability, high volatility, and thermal decomposition will require some controlled-release system such as nanotechnological formulations to optimize the action of their active ingredients [75, 76].

Thus, our findings revealed adequate insecticidal activities of clove and cinnamon essential oils against *C*. *maculatus* and demonstrated that, even at sublethal doses, these botanical compounds impaired the ability of *C*. *maculatus* to damage cowpea bean masses, which make them suitable tools that can be integrated into management programs of *C*. *maculatus*, especially for storage facilities. Further work is also needed to test the applicability and efficacy of nanofomulations of these essential oils under broader stored products conditions.

## Supporting information

S1 Data Raw data used in all the statistical analysis. (PDF)

S1 Table Summary of the non-linear regression analyses (Increase rate *r*_*i*_ and bean mass losses) of the curves shown in the Figs 1 and 2. (PDF)

S2 Table Summary of the non-linear regression analyses of average emergence curves of the curves shown in the Fig 3. (PDF)

S3 Table Summary of the non-linear regression analyses of the normalized cumulative emergence curves shown in the Fig 4. (PDF)
