# Supplementary material for: Toxicity to, oviposition and population growth impairments of Callosobruchus maculatus exposed to clove and cinnamon essential oils
Source: PLoS One. 2018 Nov 16;13(11):e0207618. doi: 10.1371/journal.pone.0207618 (PMC6239305; doi:10.1371/journal.pone.0207618)
Supplement: S1 Data — (PDF) [file pone.0207618.s001.pdf]

Cumulated emergence

	Clove					Cinnamon			
days	control	LD20	LD40	LD60	days	control	LD20	LD40	LD60
0	0	0	0	0	0	0	0	0	0
2	0,1306	0	0	0	2	6,481	0,1333	0	0
4	20,161	0,3727	0,4545	0	4	44,9726	11,8008	8,1294	0,7692
6	52,6852	6,8141	5,7576	0,7143	6	74,0883	34,1053	31,0092	6,6612
8	76,5525	25,467	31,123	22,3221	8	94,0813	73,1434	63,5628	45,9662
10	91,4659	50,9034	57,0472	52,5928	10	98,3631	86,2657	80,0771	70,882
12	97,1225	74,2978	80,1578	83,0753	12	99,5631	98,7122	94,2193	90,7429
14	98,9141	85,495	89,869	89,8445	14	99,8795	99,3228	97,2312	97,7204
16	99,622	97,6094	98,8636	98,0388	16	100	100	98,8299	98,5714
18	99,8694	98,6563	98,8636	100	18	100	100	100	100
20	99,9691	99,388	100	100					
22	100	100	100	100					

Daily emergence

				Cinnamon				
days	control	SE	LD20	SE	LD40	SE	LD60	SE
0	0	0	0	0	0	0	0	0
2	32,6	6,3922	0,4	0,4	0	0	0	0
4	190,25	7,3512	23	18,8918	10	5,3666	0,2	0,2
6	146,2	10,5707	27,8	16,7404	23,8	9,0907	1,8	1,2
8	99,6	5,8873	24,4	10,6892	25,4	6,2338	10,6	4,7074
10	21,4	2,2271	10,6	5,4918	11,6	2,5417	3,6	0,8124

12	6	1,1402	5,8	1,6248	7	1,4142	2,8	1,0677
14	1,6	0,6782	0,4	0,2449	3	1,3038	1,4	0,4
16	0,6	0,4	0,6	0,2449	1,4	0,5099	0,4	0,4
18	0	0	0	0	0,6	0,2449	0,2	0,2

Clove

days	control	SE	LD20	SE	LD40	SE	LD60	SE
0	0	0	0	0	0	0	0	0
2	0,6	0,2449	0	0	0	0	0	0
4	88	15,8032	0,6	0,6	0,4	0,4	0	0
6	135,8	19,9885	9,6	8,6348	4,6	4,3543	0,2	0,2
8	100	15,3003	17	8,2341	10	2,6458	5,4	2,1119
10	64,8	12,2368	18,8	3,8131	14,4	4,8021	7,4	2,7313
12	24,6	5,5009	16,4	2,6944	11,6	2,9086	8	2,9665
14	7,4	1,7205	7,6	2,502	4,4	1,1662	1,6	0,9274
16	3	0,8367	8	1,0488	4,2	1,3565	1,8	1,1136
18	1,2	0,5831	0,8	0,2	0	0	0,6	0,2449
20	0,4	0,2449	0,6	0,6	0,4	0,2449	0	0
22	0,2	0,2	0,6	0,4	0	0	0	0

Ovipotision

Cinnamon

Combination	Rep	Eggs
MT + FT	1	30
MT + FT	2	29
MT + FT	3	0

Clove

Combination	Rep	Eggs
TM + TF	1	2
TM + TF	2	37
TM + TF	3	0

MT + FT	4	30
MT + FT	5	31
MT + FT	6	47
MT + FT	7	36
MT + FT	8	0
MT + FT	9	0
MT + FT	10	29
MT + FT	11	37
MT + FT	12	0
MT + FT	13	1
MT + FT	14	0
MT + FT	15	1
MT + FT	16	33
MT + FT	17	50
MT + FT	18	0
MT + FT	19	3
MT + FT	20	13
MT + FT	21	30
MT + FT	22	31
MT + FT	23	46
MT + FT	24	3
MT + FT	25	13
MT + FnT	1	60
MT + FnT	2	69
MT + FnT	3	0
MT + FnT	4	51
MT + FnT	5	57
MT + FnT	6	0

TM + TF	4	55
TM + TF	5	3
TM + TF	6	0
TM + TF	7	0
TM + TF	8	36
TM + TF	9	3
TM + TF	10	8
TM + TF	11	17
TM + TF	12	50
TM + TF	13	23
TM + TF	14	29
TM + TF	15	2
TM + TF	16	5
TM + TF	17	58
TM + TF	18	20
TM + TF	19	32
TM + TF	20	0
MT + FnT	1	28
MT + FnT	2	46
MT + FnT	3	38
MT + FnT	4	74
MT + FnT	5	51
MT + FnT	6	79
MT + FnT	7	43
MT + FnT	8	34
MT + FnT	9	57
MT + FnT	10	50
MT + FnT	11	91

MT + FnT	7	0
MT + FnT	8	59
MT + FnT	9	0
MT + FnT	10	42
MT + FnT	11	0
MT + FnT	12	57
MT + FnT	13	42
MT + FnT	14	1
MT + FnT	15	0
MT + FnT	16	46
MT + FnT	17	64
MT + FnT	18	69
MT + FnT	19	41
MT + FnT	20	0
MT + FnT	21	65
MT + FnT	23	58
MT + FnT	24	55
MT + FnT	25	0
MnT + FT	1	0
MnT + FT	2	2
MnT + FT	3	10
MnT + FT	4	6
MnT + FT	5	0
MnT + FT	6	27
MnT + FT	7	6
MnT + FT	8	0
MnT + FT	9	0
MnT + FT	10	17

MT + FnT	12	57
MT + FnT	13	57
MT + FnT	14	58
MT + FnT	15	82
MT + FnT	16	0
MT + FnT	17	45
MT + FnT	18	51
MT + FnT	19	63
MT + FnT	20	55
MnT + FT	1	23
MnT + FT	2	16
MnT + FT	3	50
MnT + FT	4	0
MnT + FT	5	17
MnT + FT	6	27
MnT + FT	7	38
MnT + FT	8	22
MnT + FT	9	6
MnT + FT	10	2
MnT + FT	11	0
MnT + FT	12	0
MnT + FT	13	32
MnT + FT	14	0
MnT + FT	15	48
MnT + FT	16	36
MnT + FT	17	17
MnT + FT	18	0
MnT + FT	19	0

MnT + FT	11	2
MnT + FT	12	0
MnT + FT	13	16
MnT + FT	14	20
MnT + FT	15	0
MnT + FT	16	5
MnT + FT	17	6
MnT + FT	18	10
MnT + FT	19	9
MnT + FT	20	3
MnT + FT	21	10
MnT + FT	22	33
MnT + FT	23	5
MnT + FT	24	0
MnT + FT	25	8
Control	1	67
Control	2	0
Control	3	60
Control	4	33
Control	5	66
Control	6	0
Control	7	66
Control	8	58
Control	9	0
Control	10	41
Control	11	57
Control	12	54
Control	13	53

MnT + FT	20	24
Control	1	67
Control	2	0
Control	3	60
Control	4	33
Control	5	66
Control	6	0
Control	7	66
Control	8	58
Control	9	0
Control	10	41
Control	11	57
Control	12	54
Control	13	53
Control	14	56
Control	15	0
Control	16	0
Control	17	44
Control	18	52
Control	19	68
Control	20	62
Control	21	73
Control	22	35
Control	23	28
Control	24	27
Control	25	61
Control	26	0
Control	27	57

Control	14	56	Control	28	0
Control	15	0	Control	29	55
Control	16	0	Control	30	48
Control	17	44	Control	31	0
Control	18	52	Control	32	70
Control	19	68	Control	33	52
Control	20	62	Control	34	0
Control	21	73	Control	35	67
Control	22	35	Control	36	50
Control	23	28	Control	37	3
Control	24	27	Control	38	61
Control	25	61	Control	39	55
Control	26	0	Control	40	39
Control	27	57	Control	41	67
Control	28	0	Control	42	0
Control	29	55	Control	43	99
Control	30	48	Control	44	66
Control	31	0	Control	45	65
Control	32	70			
Control	33	52			
Control	34	0			
Control	35	67			
Control	36	50			
Control	37	3			
Control	38	61			
Control	39	55			
Control	40	39			
Control	41	67			

Control	42	0
Control	43	99
Control	44	66
Control	45	65

Instantaneous rate of population growth & bean-mass losses

clove				Cinnamon			
	Concentrations	Ri	Mass losse		Concentrations	Ri	Mass losse
Control	0	0,067603081	16,68333667	Control	0	0,073567	17,38870034
Control	0	0,069139791	17,36	Control	0	0,074997	19,46442846
Control	0	0,077258089	25,19968051	Control	0	0,075043	20,72342126
Control	0	0,052878803	7,088658147	Control	0	0,074293	19,25689173
Control	0	0,065722468	16,41046117	Control	0	0,076344	21,89562088
LC20	48,6	0,031355266	7,655993432	LC20	106,2	-0,03224	3,151805306
LC20	48,6	0,046348269	5,677728908	LC20	106,2	0,023526	4,757145713
LC20	48,6	0,014840653	1,238266427	LC20	106,2	0,00424	3,80629733
LC20	48,6	0,024780924	2,200440088	LC20	106,2	0,032528	5,358928214
LC20	48,6	0,023273755	1,675643327	LC20	106,2	0,062978	11,83160415
LC40	67,6	0,02248002	2,176517572	LC40	123	0,045254	6,549520767
LC40	67,6	0,02653161	2,66	LC40	123	0,042618	6,549520767
LC40	67,6	0,00405159	1,537847014	LC40	123	0,013669	4,05027933
LC40	67,6	-0,003611532	0,719712115	LC40	123	0,034456	5,472338726
LC40	67,6	0,032924545	3,312712034	LC40	123	0,005189	3,776978417
LC60	90,2	0,021656881	2,415651827	LC60	139,4	-0,00829	3,367204622
LC60	90,2	0,007477161	1,818545164	LC60	139,4	0,006101	3,628389155

LC60	90,2	-0,017744615	1,518784972
LC60	90,2	-0,011351681	1,28
LC60	90,2	0,003105821	1,539692062
LC80	125,8	-0,066571828	0,819672131
LC80	125,8	-0,066571828	0,719568259
LC80	125,8	-0,066571828	0,777511962
LC80	125,8	-0,030806541	0,959808038
LC80	125,8	-0,066571828	0,619504396

LC60	139,4	-0,00519	3,765690377
LC60	139,4	-0,05355	3,038784486
LC60	139,4	0,01987	4,169992019
LC80	161,4	-0,05355	2,96694544
LC80	161,4	0	3,227734608
LC80	161,4	0,01093	3,9
LC80	161,4	-0,01002	3,195078389
LC80	161,4	-0,04412	2,955271565
