# Supplementary material for: Toxicity to, oviposition and population growth impairments of Callosobruchus maculatus exposed to clove and cinnamon essential oils
Source: PLoS One. 2018 Nov 16;13(11):e0207618. doi: 10.1371/journal.pone.0207618 (PMC6239305; doi:10.1371/journal.pone.0207618)
Supplement: S1 Table — (PDF) [file pone.0207618.s002.pdf]

S1 Table. Summary of the non-linear regression analyses (increase rate r_i and bean mass losses) of the curves shown in Figures 1 and 2.

**Parameters evaluated**	**Oil**	**Model**	**Estimated parameters (±SD)**			df_{error}	F	P	R^2
			a	b	y_0				
Increase rate (r_i)	*Clove*	$y = y_0 + a*x$	-0.001 (-10^{-3} - -5.10^{-4})	-	0.1 (0.04 – 0.11)	4	54.1	0.005	0.95
(Fig. 1)	*Cinnamon*	$y = y_0 + a*x$	-0.001 (-10^{-3} – $2.\ 10^{-4}$)	-	0.1 (0.04 – 0.12)	4	30.1	0.01	0.91
Mass losses	*Clove*	$y = y_0 + a*\exp(-b*x)$	14.7 (13.1 – 16.3)	0.33 (0.02 – 0.04)	0.7 (-0.5 – 1.9)	4	110.4	0.009	0.99
(Fig. 2)	*Cinnamon*	$y = y_0 + a*x$	-0.08 (-0.09 - -0.06)	-	15.0 (12.6 –17.5)	4	147.9	0.001	0.98
