# Supplementary material for: Toxicity to, oviposition and population growth impairments of Callosobruchus maculatus exposed to clove and cinnamon essential oils
Source: PLoS One. 2018 Nov 16;13(11):e0207618. doi: 10.1371/journal.pone.0207618 (PMC6239305; doi:10.1371/journal.pone.0207618)
Supplement: S2 Table — (PDF) [file pone.0207618.s003.pdf]

S2 Table. Summary of the non-linear regression analyses (daily emergence) of the curves shown in Fig 3.

**Essential oil**	**Model**	**Dose** (µLkg^{-1})	**Estimated parameters (±SD)** *a*	*b*	x_0	df_{error}	***F***	***P***	R^2
Clove (Fig. 1 A)	y = a*exp (0.5*((x-x_0)/b)^2)	Control	133.1 (111.4 -154.8) a	2.5 (2.1 - 3.0)	6.7 (6.2 - 7.1)	11	89.7	<0.0001	0.95
		48.60	19.3 (16.24 - 22.4) b	3.3 (2.7 - 3.9)	10.1 (9.5 - 10.8)	11	76.9	<0.0001	0.94
		67.60	14.1 (12.32- 15.9) c	2.8 - (2.4 - 3.2)	10.3 (9.9 - 10.7)	11	140.7	<0.0001	0.97
		90.20	8.6 (6.9 - 10.3) d	2.3 - (1.8 - 2.8)	10.6 (10.1 -11.1)	11	63.6	<0.0001	0.93
Cinnamon (Fig. 1 B)	y = a*exp (0.5*((x-x_0)/b)^2)	Control	186.7 (142.4 - 230.1) a	2.1 (1.6 - 2.7)	5.2 (4.7 - 5.8)	9	46.6	<0.0001	0.94
		106.20	29.1 (23.9 - 36.1) b	2.5 (1.9 - 3.1)	6.5 (5.9 - 7.0)	9	57.2	<0.0001	0.94
		123.00	26.6 (22.4 - 30.7) b	2.4 (1.9 - 2.8)	7.3 (6.8 - 7.7)	9	91.9	<0.0001	0.96
		139.40	10.8 (7.8 - 13.7) c	1.2 (0.8 - 1.6)	8.3 (7.7 - 8.8)	9	30.1	0.0003	0.90
