# Supplementary material for: Toxicity to, oviposition and population growth impairments of Callosobruchus maculatus exposed to clove and cinnamon essential oils
Source: PLoS One. 2018 Nov 16;13(11):e0207618. doi: 10.1371/journal.pone.0207618 (PMC6239305; doi:10.1371/journal.pone.0207618)
Supplement: S3 Table — (PDF) [file pone.0207618.s004.pdf]

S3 Table. Summary of the non-linear regression analyses (cumulated emergence) of the curves shown in Fig 4.

Oil	Model	Dose (μLkg^{-1})	Estimated parameters (±SD) *a*	*b*	x_0	df_{error}	*F*	*P*	R^2
Clove (Fig. 2 A)	y= a/(1+exp(-(x-x_0)/b))	Control	99.2 (96.8 - 101.6) a	1.5 (1.2 -1.7) a	6.0 (5.8 - 6.3) a	11	1402.8	<0.0001	0.99
		48.60	99.4 (96.7 – 102.2) a	1.8 (1.6 - 2.0) a	10.0 (9.8 – 10.3) b	11	2271.2	<0.0001	0.99
		67.60	99.3 (96.4 - 102.2) a	1.6 (1.4 - 1.9) a	9.6 (9.3 - 9.8) b	11	1766.3	<0.0001	0.99
		90.20	98.1 (96.2 - 101.7) a	1.4 (1.1 - 1.6) a	9.8 (9.6 - 10.1) b	11	1846.5	<0.0001	0.99
Cinnamon (Fig. 2 B)	y= a/(1+exp(-(x-x_0)/b))	Control	99.5 (96.3 - 102.7) a	1.2 (1.0 - 1.5) ab	4.5 (4.2 - 4.8) a	9	763.1	<0.0001	0.99
		106.20	99.5 (96.5 – 102.5) a	1.4 (1.1 - 1.6) ab	6.8 (6.6 - 7.1) b	9	1467.2	<0.0001	0.99
		123.00	98.5 (95.1 - 101.9) a	1.5 (1.3 - 1.8) a	7.3 (7.0 - 7.6) b	9	1293.5	<0.0001	0.99
		139.40	98.6 (93.3 - 103.8) a	1.3 (1.0 - 1.7) ab	8.5 (8.1 - 8.9) c	9	656.9	<0.0001	0.99
